# Laboratory Monitoring of Nutritional Deficiencies in Children Following Restrictive Diets: A Narrative Review and Risk-Based Considerations

**DOI:** 10.3390/children13080998

**Published:** 2026-07-28

**Authors:** Dejan Dobrijević, Kristian Pastor, Mirjana Stojšić

**Affiliations:** 1 Institute for Children and Youth Health Care of Vojvodina, 21000 Novi Sad, Serbia; 2 Faculty of Medicine, University of Novi Sad, 21000 Novi Sad, Serbia; 3 Faculty of Technology Novi Sad, University of Novi Sad, 21000 Novi Sad, Serbia

**Keywords:** restrictive diets, pediatric nutrition, nutritional deficiencies, laboratory biomarkers, plant-based diet, food elimination, ketogenic diet, phenylketonuria

## Abstract

**Highlights:**

**What are the main findings?**

- Nutritional risks in children following restrictive diets depend more on the foods actually excluded, the adequacy of replacements, and adherence to supplements or medical formulas than on the dietary label alone.
- Normal laboratory values do not necessarily confirm nutritional adequacy, while abnormal findings must be interpreted in relation to inflammation, supplementation, medication, growth, and the underlying condition.

**What are the implications of the main findings?**

- Nutritional monitoring should combine dietary assessment, longitudinal growth evaluation, and targeted laboratory biomarkers selected according to individual risk.
- Universal laboratory panels and uniform testing intervals should not be applied across all restrictive dietary patterns; monitoring should be individualized within condition-specific clinical guidance.

**Abstract:**

Introduction: Restrictive diets are increasingly encountered in pediatric practice and may be adopted voluntarily or prescribed for medical conditions. Although they can support normal growth when appropriately planned, exclusion of nutritionally important foods may increase the risk of nutrient inadequacy. This narrative review examined nutritional deficiencies and laboratory monitoring in children following plant-based, food-allergy elimination, gluten-free, ketogenic, and protein-restricted diets for inherited metabolic disorders. Methods: Targeted searches of PubMed, Scopus, and Web of Science were conducted through 30 June 2026 using pediatric, diet-specific, nutritional-status, and biomarker terms. Because this was a narrative review, the literature was selected and synthesized qualitatively rather than through a formal systematic-screening process; no fixed study count, duplicate independent screening, or formal risk-of-bias assessment was performed. Professional guidelines and position papers were prioritized when discussing monitoring considerations, while pediatric studies were used to describe dietary intake, biochemical findings, clinically manifest deficiency, and growth outcomes. Results: Nutritional risks differed according to the foods or nutrients restricted. Vitamin B12 and iron were major concerns in plant-based diets, whereas cow’s milk and multiple-food elimination increased the risk of inadequate calcium, vitamin D, iodine, protein, and energy intake. Gluten-free diets were commonly associated with low fiber, iron, folate, and B-vitamin intake, particularly when refined, non-fortified products predominated. Ketogenic dietary therapy required attention to selenium, vitamin D, bone-related minerals, carnitine in selected patients, and linear growth. In phenylketonuria and related disorders, nutritional adequacy depended strongly on protein-substitute adherence and appropriate provision of essential amino acids and micronutrients. Across all dietary patterns, laboratory results required interpretation in relation to dietary intake, growth, supplementation, inflammation, medication, and the underlying condition. Across dietary patterns, inadequate intake, biochemical abnormalities, clinically manifest deficiency, and impaired growth were considered related but distinct outcomes. Conclusions: Nutritional monitoring should be individualized and based on the actual dietary restriction and clinical risk. The principal contribution of this review is a practical, risk-based framework that links the specific dietary restriction and adequacy of replacement foods with growth, symptoms, supplementation, and targeted laboratory biomarkers.

## 1. Introduction

Restrictive dietary patterns are increasingly encountered in pediatric practice. Some are adopted voluntarily for ethical, environmental, cultural, religious, or perceived health-related reasons, whereas others are medically prescribed for the management of food allergy, celiac disease, drug-resistant epilepsy, or inherited metabolic disorders. Although these dietary patterns differ substantially in their purpose and composition, they share the systematic exclusion or marked restriction of nutritionally important foods, food groups, macronutrients, or specific amino acids. The nutritional consequences depend not only on what is excluded but also on the quality of replacement foods, use of fortified products or medical formulas, supplementation, adherence, and access to professional dietary support. Restrictive diets should therefore not be considered inherently inadequate: some are medically essential, while others can support normal growth when appropriately planned and monitored [1,2,3].

Children are particularly vulnerable to dietary inadequacy because nutrient requirements must support continuous growth and development as well as normal physiological function. The risk is greatest during infancy, early childhood, puberty, and other periods of rapid growth, when requirements relative to body size are high and nutrient reserves may be limited. Inadequate intake of energy, protein, essential amino acids, iron, vitamin B12, folate, vitamin D, calcium, iodine, zinc, selenium, and essential fatty acids may impair growth, hematopoiesis, neurodevelopment, thyroid function, or skeletal mineralization. However, the nutrients most likely to be affected vary according to the dietary pattern, the child’s age, the duration and degree of restriction, and the nutritional quality of the remaining diet [1,3].

The present review focuses on five clinically relevant categories of sustained dietary restriction in children: vegetarian and vegan diets, food-allergy elimination diets, the gluten-free diet, ketogenic dietary therapy, and therapeutic diets for selected inherited metabolic disorders. These categories were selected because they involve predictable restrictions of nutritionally important foods or nutrients and may therefore justify targeted nutritional and laboratory assessment. Diets excluding animal-source foods require particular attention to vitamin B12 and may also compromise iron, zinc, calcium, iodine, vitamin D, and long-chain omega-3 fatty-acid status. Elimination of cow’s milk, wheat, eggs, fish, or multiple major allergens creates different nutrient gaps depending on the foods removed and the suitability of their replacements. A gluten-free diet may provide insufficient fiber, iron, folate, and other B vitamins when fortified wheat products are replaced predominantly with refined, non-fortified alternatives. Ketogenic dietary therapy may reduce the intake of several vitamins, minerals, and trace elements, whereas diets for inherited metabolic disorders may depend heavily on specialized formulas and carefully controlled provision of protein and essential amino acids [1,4,5].

Nutritional deficiencies may develop gradually and remain clinically silent during their early stages. Growth faltering, pallor, fatigue, bone pain, skin changes, or neurological symptoms may appear only after nutrient stores have been substantially depleted and are often nonspecific. Conversely, normal growth, body mass index, or physical examination does not exclude an isolated or subclinical deficiency. Dietary history, assessment of replacement foods and supplements, longitudinal anthropometry, and clinical examination therefore remain the foundation of nutritional evaluation. Laboratory biomarkers can provide complementary information about nutrient exposure, circulating concentrations, body stores, metabolic availability, or functional consequences of inadequate intake [3,6].

The clinical interpretation of nutritional biomarkers is nevertheless complex. A measured concentration may reflect recent intake, tissue reserves, metabolic regulation, or a functional consequence of deficiency, and these dimensions are not interchangeable. Results may also be influenced by inflammation, infection, hydration, organ function, medication use, recent supplementation, age-related physiological variation, and the timing and conditions of specimen collection. For example, ferritin may remain normal or increase during inflammation, normal serum calcium does not demonstrate adequate calcium intake, and normal hematological indices do not exclude functional vitamin B12 deficiency. Laboratory testing should therefore be selected according to a plausible dietary or clinical risk and interpreted together with dietary intake, supplement adherence, growth, symptoms, and the underlying medical condition [6,7].

Recommendations for nutritional laboratory assessment remain fragmented across dietary patterns and clinical conditions. Broad biochemical panels are not justified for every asymptomatic child, and no single set of tests or monitoring interval can be applied universally. The most informative approach is therefore risk-based: the choice of biomarkers should reflect the exact foods or nutrients restricted, the child’s age and growth phase, the duration and severity of restriction, the nutritional adequacy of substitutes or medical formulas, supplementation practices, previous laboratory findings, and the presence of clinical symptoms or additional risk factors [8,9].

This narrative review aims to summarize the principal nutritional inadequacies and deficiencies associated with vegetarian and vegan diets, food-allergy elimination diets, the gluten-free diet, ketogenic dietary therapy, and therapeutic diets for selected inherited metabolic disorders in children. It also discusses the clinical utility and interpretative limitations of selected laboratory biomarkers and proposes a practical structure for risk-based nutritional assessment. By considering these different dietary patterns within a shared framework, the review highlights both general principles of assessment and diet- or condition-specific nutritional concerns. Particular emphasis is placed on the foods and nutrients actually restricted, the adequacy of replacement foods, fortified products, supplements or medical formulas, longitudinal growth, clinical findings, and factors that may influence biomarker interpretation. The proposed framework is intended as a practical synthesis of the available evidence and should not be interpreted as a universal screening protocol, a set of formally graded clinical recommendations, or a source of fixed monitoring intervals. Restrictive eating disorders, avoidant/restrictive food intake disorder, sensory-based food selectivity, autism-related feeding difficulties, short-term diagnostic elimination diets, and cultural or religious food practices without a clearly defined nutritional restriction remain outside the scope of this review.

## 2. Methods

### 2.1. Literature Search Strategy

This narrative review synthesizes evidence on nutritional inadequacies, nutrient deficiencies, and their assessment in children following five restrictive dietary patterns: vegetarian and vegan diets, food-allergy elimination diets, the gluten-free diet, ketogenic dietary therapy, and therapeutic diets for phenylketonuria and selected protein-restricted inherited metabolic disorders. A systematic review methodology was not pursued because the primary aim was to integrate heterogeneous evidence from clinical guidelines, position papers, reviews, observational studies, and selected clinical reports and to develop a practical, risk-based framework for nutritional assessment.

Targeted and iterative literature searches were conducted in PubMed, Scopus, and Web of Science up to 30 June 2026. No lower publication-date limit was applied. Search terms were adapted to each of the five dietary categories and their principal nutritional concerns rather than applied as a single prespecified database-specific search string. Search terms combined pediatric descriptors [“child”, “children”, “infant”, “adolescent”, or “pediatric”) with diet-specific terms, including “vegetarian diet”, “vegan diet”, “plant-based diet”, “food allergy”, “elimination diet”, “gluten-free diet”, “celiac disease”, “ketogenic diet”, “phenylketonuria”, “inherited metabolic disorder”, “tyrosinemia”, “organic aciduria”, and “urea-cycle disorder”. These were combined with terms related to nutritional status and assessment, including “nutrient deficiency”, “micronutrient deficiency”, “growth”, “biomarker”, “laboratory assessment”, “vitamin B12”, “iron”, “folate”, “vitamin D”, “calcium”, “zinc”, “selenium”, “iodine”, “carnitine”, “fatty acids”, and “amino acids”. Searches were supplemented by manual screening of the reference lists of relevant publications. Titles and abstracts were assessed for relevance and potentially relevant full texts were evaluated against the predefined scope of the review. Publications were retained when they directly addressed one of the included dietary patterns and provided information on pediatric dietary intake, growth, biochemical nutrient status, clinically manifest deficiency, or laboratory assessment. As this was a narrative review, no formal duplicate independent screening or detailed exclusion log was undertaken.

Peer-reviewed English-language studies involving children or adolescents were prioritized. Professional-society guidelines, position papers, and systematic reviews were used primarily to identify clinically relevant nutritional risks and monitoring recommendations, while pediatric primary studies were used to describe dietary intake, biochemical findings, growth outcomes, and the influence of supplementation or medical-formula adherence. Case reports were considered selectively when they illustrated severe or uncommon nutritional deficiencies. Adult-only studies, animal and in vitro studies, and publications focused exclusively on disease control, treatment efficacy, or metabolic complications unrelated to nutritional deficiency were excluded.

Restrictive eating disorders, avoidant/restrictive food intake disorder, sensory-based food selectivity, autism-related feeding difficulties, short-term diagnostic diets, and cultural or religious food practices without a defined nutritional restriction were outside the scope of this review.

### 2.2. Evidence Synthesis

Evidence was synthesized narratively because of substantial heterogeneity in dietary definitions, age groups, supplementation practices, laboratory methods, and reported outcomes. Dietary inadequacy and biochemical deficiency were considered distinct outcomes, and laboratory findings were interpreted in relation to dietary intake, growth, supplementation, inflammation, medication use, and the underlying clinical condition.

The proposed assessments were not treated as formal recommendations of equal evidentiary strength. Where condition-specific professional guidelines or position papers were available, their recommendations informed the framework. In areas without sufficiently specific professional guidance, the tables reflect the authors’ qualitative synthesis of pediatric evidence, established principles of nutritional biomarker interpretation, and the biological plausibility of nutrient risk arising from the foods or nutrients excluded.

When findings were inconsistent, greater interpretative weight was given to recent professional-society guidelines and position papers, systematic reviews, longitudinal pediatric studies, and studies with clearly defined dietary patterns, duration of restriction, supplementation practices, and clinically interpretable outcomes. Small cross-sectional studies and case reports were used primarily to illustrate possible or severe nutritional risks and were not used alone to support general monitoring approaches. A nutritional concern or biomarker was included in the proposed framework when it was supported by condition-specific guidance, repeatedly identified in pediatric evidence, or had a direct and biologically plausible relationship with the food or nutrient excluded. When uncertainty remained, this was stated explicitly, and routine testing was not proposed.

The assessment tables were developed as a pragmatic synthesis of the included literature. They link each dietary pattern with its principal nutritional concerns, proposed assessment, and key interpretative considerations. The tables are intended as a risk-based clinical framework and should not be interpreted as universally endorsed screening panels or fixed monitoring protocols.

As this was a narrative review, no formal risk-of-bias assessment or grading of the strength of individual recommendations was performed.

## 3. Restrictive Dietary Patterns Associated with Nutritional Deficiencies in Children

### 3.1. Plant-Based Diets

Plant-based diets encompass several dietary patterns that differ in the extent to which foods of animal origin are excluded. Lacto-ovo-vegetarian diets exclude meat and fish but include dairy products and eggs, whereas vegan diets exclude all animal-derived foods. More restrictive variants, such as raw vegan and fruitarian diets, impose additional limitations and should not be considered nutritionally equivalent to conventional vegetarian or vegan diets. Families may adopt these patterns for ethical, environmental, cultural, religious, or perceived health-related reasons, and children commonly follow the dietary practices selected by their parents. The nutritional implications therefore depend not only on the dietary label but also on the child’s age, the degree and duration of restriction, food variety, use of fortified products, supplementation, and access to professional dietary counselling [10,11,12].

Plant-based diets should not be regarded as inherently deficient. A varied lacto-ovo-vegetarian diet generally carries a lower nutritional risk because eggs and dairy products provide nutrients that are absent or less available in exclusively plant-based foods. In contrast, a vegan diet depends more heavily on carefully selected foods, fortification, and supplementation, particularly for vitamin B12. Nutritional vulnerability is greatest in infancy and early childhood because of rapid growth, high nutrient requirements relative to body size, limited body stores, and low gastric capacity. Recent systematic reviews have not demonstrated consistent differences in average weight, height, or body mass index between vegetarian and omnivorous children, although individual studies have reported lower height or BMI z-scores, greater prevalence of underweight, or lower bone mineral content in some vegan groups. Interpretation remains limited by predominantly cross-sectional designs, small vegan samples, inconsistent dietary definitions, variable supplementation, and repeated analyses of the same cohorts. Current evidence is therefore insufficient to confirm that a strictly vegan diet consistently supports normal growth, supporting regular assessment of dietary intake, growth, and nutritional status [12,13,14].

#### 3.1.1. Nutritional Risks

Plant-based diets are commonly rich in fiber and relatively low in energy density. Although recent pediatric studies generally report similar total energy intake in vegetarian, vegan, and omnivorous children, high intake of bulky, low-energy foods may limit energy consumption in infants, young children, and selective eaters. Protein intake is usually quantitatively adequate, but plant proteins may have lower digestibility and a less favorable indispensable amino acid profile than animal proteins. Cereals tend to be relatively low in lysine, whereas legumes may provide less methionine. A varied diet containing legumes, soy foods, cereals, nuts, and seeds can provide complementary amino acid profiles, but the risk of inadequate protein supply increases when total energy intake is low, food variety is poor, or additional dietary restrictions are present. The most recent ESPGHAN position paper consequently recommends assessing both the quantity and quality of protein intake individually in vegan children [12,14,15].

Vitamin B12 is the most clearly established nutrient of concern in vegan diets. Reliable naturally occurring plant sources are lacking, and algae, fermented foods, and similar products cannot be considered dependable substitutes because their vitamin B12 content and biological activity are variable. Adequacy therefore requires regular consumption of fortified foods and an age-appropriate supplement. Risk is lower, but not absent, in lacto-ovo-vegetarian children, particularly when dairy and egg consumption is limited. Infants born to or breastfed by mothers with inadequate vitamin B12 status are especially vulnerable because neonatal stores and breast-milk concentrations depend on maternal status. Deficiency may present with feeding difficulties, growth faltering, hypotonia, developmental regression, anemia, or neurological damage. In addition, high folate intake may attenuate the macrocytic hematological features of vitamin B12 deficiency, meaning that normal hemoglobin or mean corpuscular volume does not reliably exclude functional deficiency [11,12,16].

Available pediatric studies demonstrate that vitamin B12 status is strongly influenced by supplementation. Unsupplemented or inadequately supplemented vegan children have shown lower circulating vitamin B12 or holotranscobalamin and higher concentrations of functional markers such as methylmalonic acid. Conversely, adequately supplemented children may have concentrations comparable with, or considerably higher than, those of omnivorous children. Therefore, the dietary category alone is an inadequate predictor of vitamin B12 status; the formulation, dose, frequency, and adherence to supplementation should always be documented [12,14].

Iron intake may be quantitatively adequate or even higher in vegetarian and vegan children, but this does not necessarily indicate adequate iron status. Plant foods provide non-heme iron, which has lower and more variable bioavailability than heme iron. Its absorption is inhibited by phytates and polyphenols and enhanced by vitamin C and certain food-processing methods, including soaking, germination, and fermentation. Recent evidence indicates that vegetarian and vegan children may have lower ferritin concentrations and a higher prevalence of depleted iron stores, even when mean hemoglobin remains within age-appropriate reference limits. Evidence for a consistently higher prevalence of overt anemia is less conclusive. Particular attention is warranted in infants after the first six months of life, children during rapid growth, menstruating adolescents, selective eaters, and children with limited intake of iron-rich or fortified foods [11,12,14,16].

Calcium and vitamin D require attention when dairy products are excluded. Calcium intake depends on the use of fortified plant-based alternatives, calcium-set tofu, selected low-oxalate vegetables, or supplements. The nutrient composition of plant-based beverages varies substantially, and these products should not be assumed to be nutritionally equivalent to breast milk, infant formula, or cow’s milk. Vitamin D inadequacy is common throughout the general pediatric population and is affected by supplementation, season, latitude, skin pigmentation, clothing, and sunlight exposure. Nevertheless, vegan children have fewer dietary sources, and some studies have reported lower serum 25-hydroxyvitamin D concentrations despite supplementation. Evidence regarding skeletal outcomes remains limited, although lower bone mineral content and altered bone-turnover markers have been observed in some vegan or vegetarian cohorts. These findings do not establish universal skeletal impairment but support targeted evaluation in children with inadequate calcium intake, inconsistent vitamin D supplementation, poor growth, bone pain, fractures, or other risk factors [12,14,17].

The status of zinc, iodine, and selenium is more difficult to evaluate. Legumes, whole grains, nuts, and seeds provide zinc, but its absorption may be reduced by dietary phytates. Pediatric studies have reported inconsistent differences in zinc intake and circulating concentrations, and clinically evident deficiency appears uncommon in well-characterized cohorts. Iodine intake may be low when fish, dairy products, and eggs are excluded and iodized salt or fortified products are not used regularly. Moreover, the iodine content of plant foods varies with soil and agricultural conditions. Some recent cohorts have reported a higher prevalence of low urinary iodine concentrations in vegan children, although the available evidence remains limited. Selenium intake is similarly influenced by soil content and food origin, and lower intake has been reported in some vegetarian and vegan groups. These nutrients should therefore be assessed according to dietary exposure and clinical risk rather than through indiscriminate routine testing [11,12,14].

Plant-based diets also tend to provide little preformed eicosapentaenoic acid and docosahexaenoic acid, because fish is excluded and endogenous conversion from alpha-linolenic acid is limited. Vegan children may consume adequate or high amounts of alpha-linolenic acid from seeds, nuts, and vegetable oils, but this does not necessarily ensure adequate long-chain omega-3 fatty-acid status. Current evidence does not support routine measurement of fatty-acid profiles in otherwise healthy children; however, dietary intake should be reviewed, and an age-appropriate algal DHA source may be considered, particularly during infancy and early childhood. Riboflavin, vitamin A, and choline may also warrant attention in poorly planned or highly restrictive diets, although the pediatric evidence is currently insufficient to justify universal biochemical screening [12,16,17].

#### 3.1.2. Laboratory Assessment and Monitoring

Laboratory testing should complement, rather than replace, dietary history, longitudinal growth assessment, and clinical examination. Evaluation should establish the exact dietary pattern, age at initiation, duration and severity of restriction, use of fortified products, supplement formulation and adherence, breastfeeding history and maternal vitamin B12 status in infants, food selectivity, growth trajectory, pubertal stage, menstrual blood loss, and symptoms potentially associated with deficiency. A broad laboratory panel is not automatically required in every asymptomatic child consuming a varied lacto-ovo-vegetarian diet. Testing should be individualized according to the degree of restriction, age, quality of dietary planning, supplementation, growth, and clinical findings (Table 1) [11,12,16].

The 2025 ESPGHAN position paper recommends monitoring vitamin B12 metabolic parameters in vegan children during periods of rapid growth and when supplementation or intake may be inadequate. Iron and vitamin D status should be assessed when dietary history, symptoms, growth abnormalities, or poor adherence indicate increased risk. No sufficiently evidence-based universal testing interval has been established. Accordingly, the table should be interpreted as a risk-based framework rather than as a mandatory panel for every vegetarian or vegan child [12,14,16].

A child consuming a varied lacto-ovo-vegetarian diet, growing appropriately, and receiving reliable nutrient sources may not require investigations beyond usual pediatric care. A lower threshold for targeted laboratory testing is appropriate in vegan infants and children without documented supplementation, those with highly selective or additionally restricted diets, and children presenting with poor growth, pallor, fatigue, developmental concerns, neurological symptoms, bone complaints, or inconsistent adherence. Laboratory findings should always be interpreted together with dietary intake, supplementation history, anthropometry, development, and clinical examination. Vegetarian and vegan diets can support adequate growth and nutritional status when they are varied, age-appropriate, and accompanied by reliable vitamin B12 supplementation and other fortified foods or supplements according to individual need [18,19].

### 3.2. Elimination Diets in Children with Food Allergy

Elimination of the causative food remains a central component of the management of confirmed food allergy. However, the nutritional consequences arise primarily from the exclusion of nutritionally important foods and their inadequate replacement, rather than from the diagnosis of food allergy itself. The degree of risk depends on the child’s age, the number and nutritional importance of the foods removed, the duration of restriction, the quality of substitute products, supplementation, feeding difficulties, coexisting gastrointestinal or atopic disease, and access to specialist dietary counselling. Infants and young children are particularly vulnerable because of rapid growth, high nutrient requirements relative to body size, and dependence on a limited number of staple foods [20,21,22].

Elimination should therefore be limited to foods for which avoidance is clinically justified. Inappropriate restrictions may result from unconfirmed diagnoses, interpretation of sensitization tests without a compatible clinical history, parental anxiety, or prolonged continuation of a diagnostic elimination diet after it has ceased to provide clinical benefit. Periodic reassessment of allergic status and supervised reintroduction, when appropriate, can reduce unnecessary restriction and improve dietary variety. Professional dietary support is especially important when milk, wheat, egg, soy, or several food groups are excluded, because these foods provide substantial amounts of energy, protein, vitamins, and minerals and are widely distributed in the usual pediatric diet [21,22,23].

Growth impairment is not inevitable in children following elimination diets. Some studies have reported lower weight-for-age or height-for-age indices in food-allergic children, particularly when several foods were excluded, whereas others have shown that adequate substitution, supplementation, and regular physician–dietitian follow-up can preserve nutrient intake and laboratory status. In the study by Flammarion et al. [24]., children with food allergy had lower weight-for-age and height-for-age z-scores than matched controls, and those avoiding three or more foods were smaller than children avoiding one or two. Conversely, Berry et al. [25] found no significant additional deterioration in growth or laboratory nutritional markers in children eliminating cow’s milk and wheat compared with those eliminating milk alone when the diets were carefully supervised. These findings indicate that the number of exclusions is an important risk marker, but the nutritional quality of the resulting diet may be more decisive than the restriction itself [24,25].

#### 3.2.1. Cow’s Milk Elimination

Cow’s milk elimination deserves particular attention because milk and dairy products contribute substantially to energy, high-quality protein, calcium, phosphorus, vitamin D where fortification is used, vitamin B12, riboflavin, and iodine. The nutritional effect of milk avoidance depends strongly on age and on the type of replacement. In non-breastfed infants with confirmed cow’s milk allergy, nutritionally complete hypoallergenic formulas are required, whereas ordinary commercial plant-based beverages are not nutritionally equivalent to breast milk or infant formula. In older children, fortified plant-based beverages may contribute to calcium and vitamin D intake, but their protein and energy contents vary considerably, and some products function mainly as culinary substitutes rather than as true nutritional replacements [21,22,23].

Calcium and vitamin D are the principal micronutrients of concern. Low calcium intake may occur when dairy products are removed without regular use of calcium-fortified alternatives or supplementation. Vitamin D status is additionally influenced by sunlight exposure, season, skin pigmentation, supplementation, and national fortification practices. Severe cases of hypocalcaemia and nutritional rickets have been described in children following poorly supervised milk-free or broadly restrictive diets, although these reports represent the extreme end of the clinical spectrum rather than the expected outcome of an appropriately managed elimination diet [20,21,26].

Interpretation of calcium status requires caution. Serum calcium is tightly regulated and may remain within the reference interval despite chronically inadequate intake, because parathyroid hormone and skeletal calcium mobilization maintain extracellular concentrations. Therefore, normal serum calcium should not be interpreted as evidence of adequate calcium consumption. Dietary assessment and review of supplementation are primary, while serum phosphate, alkaline phosphatase, parathyroid hormone, and 25-hydroxyvitamin D provide additional information when deficiency, poor growth, bone pain, fractures, or impaired mineralization are suspected.

Protein and energy intake may also become inadequate, particularly in infants and young children when cow’s milk is replaced with beverages that contain little protein or energy. Flammarion et al. [24] reported lower protein and food-derived calcium intake in children with cow’s milk allergy, whereas total intake improved when appropriate milk substitutes and calcium supplements were included. Severe protein-energy malnutrition, hypoalbuminaemia, and kwashiorkor have been reported after use of nutritionally unsuitable substitutes such as rice beverages as the main milk replacement, but such outcomes are mainly associated with unsupervised or highly unbalanced diets [21,24,26].

Iodine may represent an additional concern because dairy products are an important source in several countries. Low urinary iodine concentrations have been reported in some young children avoiding cow’s milk, particularly when iodized salt, fortified foods, or iodine-containing formulas and supplements are not used. However, iodine status depends strongly on regional food composition and national fortification practices and should therefore be assessed within the local dietary context rather than assumed to be low in every child [20,21,22].

#### 3.2.2. Multiple-Food Elimination

The nutritional risk generally increases as more foods or food groups are removed, particularly when the excluded foods normally serve as major sources of energy, protein, or micronutrients. Simultaneous elimination of milk and wheat is especially challenging because milk contributes protein and calcium, while wheat and other grains provide energy, complex carbohydrates, fiber, iron, folate, thiamine, riboflavin, and other B vitamins. Exclusion of egg, soy, nuts, fish, or legumes may further reduce dietary variety and limit sources of protein, essential fatty acids, iron, zinc, selenium, vitamin D, and vitamin B12, depending on the remaining diet [21,22,23].

Iron deficiency is of particular relevance when the resulting diet contains limited meat, fortified cereals, legumes, or other iron-rich foods. The risk may be amplified in infants, rapidly growing children, menstruating adolescents, and patients with selective eating [27]. However, reduced dietary intake should not be equated automatically with biochemical deficiency. Available studies have not consistently demonstrated lower hemoglobin or ferritin in all food-allergic children, especially when diets are supervised and supplementation is provided. In the recent study by Jasielska et al. [28], children assessed at the time of food-allergy diagnosis showed no significant differences from controls in hemoglobin, ferritin, albumin, total protein, or 25(OH)D, despite a higher prevalence of underweight among children younger than 30 months. Since these children were evaluated before permanent elimination, the findings mainly show that overt biochemical abnormalities may be absent at baseline and support individualized follow-up rather than automatic assumptions of deficiency [27,28].

Children following multiple-food elimination diets may also develop feeding difficulties. Fear of allergic reactions, painful previous experiences, delayed introduction of new textures, parental caution, and a reduced number of repeated food exposures can contribute to food aversion or avoidant/restrictive eating patterns. These problems can further narrow dietary variety and reduce total energy and nutrient intake independently of the foods that must medically be avoided. Nutritional review should therefore include not only the list of allergens but also meal structure, accepted foods, texture progression, eating behavior, and family anxiety related to feeding [20,21,22].

#### 3.2.3. Laboratory Assessment and Monitoring

Laboratory testing should complement dietary history, anthropometry, and clinical examination. The initial evaluation should document the exact foods eliminated, duration and strictness of avoidance, use and composition of substitutes, supplementation, growth trajectory, gastrointestinal symptoms, feeding difficulties, pubertal stage, menstrual blood loss, and signs that may suggest deficiency. Particular attention is warranted in children younger than two years, those excluding cow’s milk or several food groups, children with poor growth or food selectivity, and patients whose diets are not supervised by a pediatric dietitian.

No universal laboratory panel has been established for every child with food allergy. Biomarkers should be selected according to the nutrients normally supplied by the excluded foods and the individual clinical risk (Table 2) [20,22].

For children avoiding cow’s milk, serum 25(OH)D is the most informative routine marker of vitamin D status. Calcium, phosphate, alkaline phosphatase, and parathyroid hormone are more appropriate when calcium intake is low, vitamin D deficiency is identified, growth is impaired, or bone disease is suspected. Because calcium homeostasis maintains serum calcium within a narrow range, dietary intake and supplement adherence remain essential components of assessment [29,30].

When iron deficiency is possible, CBC and ferritin provide a practical initial evaluation. Ferritin should be interpreted alongside clinical evidence of infection or inflammation and, when appropriate, CRP. Transferrin saturation or soluble transferrin receptor may help when ferritin and hematological findings are discordant. Measurement of serum iron alone is not recommended as an adequate assessment of iron status because of its biological variability and limited specificity.

Assessment of protein adequacy should rely primarily on dietary intake, weight gain, linear growth, and clinical examination. Albumin, prealbumin, total protein, and retinol-binding protein may be altered in severe nutritional compromise, but they are not specific markers of dietary protein intake and are influenced by inflammation, hepatic synthesis, renal losses, and hydration. Although lower RBP was observed in the study by Jasielska et al. [28], this isolated finding is insufficient to support routine RBP screening in all food-allergic children.

Children consuming a nutritionally complete replacement diet, growing normally, and receiving appropriate supplementation may not require broad biochemical investigation. A lower threshold for targeted testing is appropriate when cow’s milk or multiple staple foods are excluded, when suitable substitutes are absent, or when there is poor growth, pallor, fatigue, feeding difficulty, bone pain, fractures, developmental concern, or prolonged unsupervised restriction. Laboratory findings should always be interpreted together with dietary exposure, supplementation, anthropometry, and the clinical course. Medically indicated elimination diets can be implemented safely when unnecessary restrictions are avoided, nutritionally appropriate substitutes are used, and growth, dietary intake, and supplementation are reviewed regularly.

### 3.3. Gluten-Free Diet

A gluten-free diet (GFD) requires the long-term exclusion of wheat, rye, and barley and is essential for children with celiac disease and, in selected circumstances, other medically confirmed gluten-related disorders. From a nutritional perspective, the principal concern is not the absence of gluten itself but the removal of commonly consumed grain products and their replacement with foods that may not provide an equivalent amount of fiber, iron, folate, B vitamins, or other micronutrients. Deficiencies identified at diagnosis may initially reflect intestinal damage and malabsorption, whereas abnormalities that persist or newly develop after mucosal recovery may be related to the composition and overall quality of the GFD [31,32,33].

The nutritional adequacy of a GFD depends strongly on the foods used to replace gluten-containing grains. A diet based on naturally gluten-free foods—including potatoes, legumes, fruits, vegetables, dairy products, meat, fish, eggs, nuts, seeds, and nutrient-dense grains or pseudocereals—can be nutritionally complete. In contrast, frequent reliance on refined commercial gluten-free products may result in a diet with less fiber and micronutrients and, in some cases, more fat, sugar, and rapidly digestible starch. The most recent systematic review of pediatric studies found substantial heterogeneity, but persistent inadequacies in several nutrients were common during both short- and long-term GFD adherence [33,34].

#### 3.3.1. Nutritional Characteristics and Long-Term Risks

Dietary fiber is one of the most consistent concerns. Wheat-based breads and cereals are important sources of fiber in many children’s diets, while numerous gluten-free products are made predominantly from refined rice flour, corn starch, potato starch, or tapioca. Children who consume few whole grains, legumes, fruits, and vegetables may therefore have a low fiber intake despite adequate total energy intake. Fiber inadequacy may contribute to constipation and a less favorable overall dietary pattern, but it cannot be assessed through a routine laboratory biomarker. Dietary history, food records, stool pattern, and the types of grain substitutes consumed are therefore more informative than blood testing [31,33,35].

Iron remains the micronutrient of greatest practical relevance. Iron deficiency may be present before GFD initiation because of malabsorption, but low intake can persist after treatment if fortified wheat products are replaced with non-fortified gluten-free alternatives. Fortification policies differ among countries, and gluten-free flours and breads are not always enriched to the same extent as conventional products. Pediatric studies following children for several years have reported persistent or newly developed low iron and ferritin concentrations, including in children whose celiac antibodies had normalized. Nevertheless, low dietary iron intake does not invariably produce anemia, and the contribution of menstruation, inflammation, selective eating, rapid growth, and other causes must also be considered [32,33,36].

Folate and other B vitamins may also be insufficient. Refined gluten-free products frequently contain less folate, thiamine, riboflavin, niacin, and vitamin B6 than fortified wheat-based foods. The 2025 systematic review found that folate inadequacy was particularly persistent, while lower intakes of vitamins B1, B2, B3, and B6 were reported in several pediatric studies. However, biochemical deficiencies were less consistently demonstrated than low dietary intake, and many affected children still had concentrations within laboratory reference intervals. Vitamin B12 is a more selective concern because its principal dietary sources—meat, eggs, dairy products, and fortified foods—are not inherently excluded by a GFD. Low B12 should therefore prompt assessment of the overall diet, residual malabsorption, additional restrictions, or other clinical causes rather than being attributed automatically to gluten exclusion [33,35,37].

Vitamin D is relevant because of its role in bone mineralization, but low status is not specific to the GFD. Several studies have found inadequate dietary intake or low serum 25-hydroxyvitamin D in children following a GFD, although similar abnormalities are also common in healthy pediatric populations. Season, sunlight exposure, skin pigmentation, supplementation, obesity, and regional food-fortification practices must therefore be considered. Calcium and magnesium intake may also be suboptimal, particularly when lactose intolerance or unnecessary dairy avoidance accompanies the GFD. A normal serum calcium concentration does not confirm adequate calcium intake or normal bone mineralization because serum calcium is tightly homeostatically regulated [32,33,35].

Available evidence also shows that nutrient intake and biochemical status should not be treated as equivalent outcomes. Ballestero Fernández et al. [35] reported lower intakes of iron, folate, calcium, magnesium, and several B vitamins in children following a GFD for more than one year, while most measured biochemical markers remained within reference ranges. Similarly, the systematic review by Papoutsaki et al. [33] concluded that dietary inadequacies were more consistently observed than laboratory-confirmed deficiencies. These findings support dietary correction before overt deficiency develops and argue against assuming that normal laboratory results necessarily indicate a nutritionally optimal diet [33,35].

#### 3.3.2. Importance of Dietary Quality

The principal objective of dietary counselling should be to achieve a nutritionally adequate GFD rather than simply to eliminate gluten. Greater use of naturally gluten-free whole foods, certified gluten-free oats where appropriate, legumes, and pseudocereals such as buckwheat, quinoa, amaranth, and millet may improve fiber, iron, folate, magnesium, and B-vitamin intake. Fortified gluten-free products can also be useful, but their nutrient composition should be checked individually because fortification is inconsistent. Children and caregivers should be taught to evaluate not only the presence of gluten but also fiber, protein, iron, folate, vitamin D, calcium, sugar, and saturated-fat content [31,33,34].

Professional dietary follow-up remains important even after symptoms resolve and serological markers normalize. Kreutz et al. [32] observed that some deficiencies developed during follow-up after previously normal results and that deficiency rates were not substantially different when analyses were restricted to children with normalized celiac antibodies. This suggests that successful control of the underlying disease does not guarantee nutritional adequacy and that long-term counselling should address food quality, fortification, supplementation, and changes in requirements during growth and adolescence [32].

#### 3.3.3. Laboratory Assessment and Monitoring

Laboratory assessment should be interpreted together with dietary intake, growth trajectory, symptoms, supplementation, adherence to the prescribed diet, and the results of previous testing. Broader testing may be justified at the beginning of dietary treatment because pre-existing deficiencies can reflect malabsorption. During long-term follow-up, testing should become more targeted and should focus on previously documented deficiencies, poor dietary quality, rapid growth, menstruation, low supplement adherence, persistent symptoms, or abnormal anthropometry. Current evidence does not support an identical broad micronutrient panel for every asymptomatic child at every visit (Table 3) [32,33].

CBC and ferritin constitute the most practical initial evaluation when iron deficiency is suspected. Ferritin should not be interpreted in isolation because it can rise during inflammation, whereas serum iron alone is too variable to serve as a reliable marker of iron status. Folate and B12 testing is reasonable in children with macrocytosis, anemia, neurological symptoms, poor dietary variety, several simultaneous restrictions, or previously abnormal results. Measurement of 25(OH)D is most relevant in children with low intake, limited sunlight exposure, impaired growth, bone pain, fractures, or other risk factors for poor bone health [38,39].

The nutritional risk associated with a GFD is determined less by gluten exclusion itself than by the quality of the foods used to replace gluten-containing grains. A well-planned diet based on naturally nutrient-rich foods may meet pediatric requirements, whereas prolonged dependence on refined, non-fortified gluten-free products can contribute to inadequate intake of fiber, iron, folate, B vitamins, and vitamin D. Dietary assessment, longitudinal growth monitoring, and selective laboratory testing therefore provide a more rational approach than routine indiscriminate screening. A gluten-free diet can be nutritionally adequate when it is based predominantly on naturally nutrient-dense gluten-free foods and appropriately fortified products rather than on refined replacement products alone [40,41,42].

### 3.4. Ketogenic Diet

Ketogenic dietary therapy is a high-fat, very-low-carbohydrate intervention used primarily in children with drug-resistant epilepsy and in selected inherited metabolic disorders, particularly glucose transporter type 1 deficiency syndrome and pyruvate dehydrogenase deficiency. Several variants are available, including the classic ketogenic diet, medium-chain triglyceride diet, modified Atkins diet, and low-glycemic-index treatment. These approaches differ in carbohydrate restriction, ketogenic ratio, protein allowance, energy prescription, and permitted food variety and should therefore not be considered nutritionally equivalent. Most pediatric evidence relates to medically prescribed diets implemented and monitored by multidisciplinary teams and cannot be directly extrapolated to unsupervised ketogenic diets adopted for weight control or other nonmedical purposes [43,44,45].

Nutritional risk arises from the marked restriction of cereals, fruits, legumes, milk, and other carbohydrate-containing foods, together with controlled energy or protein intake in some protocols. As a result, ketogenic diets may provide insufficient amounts of several vitamins, minerals, trace elements, and fiber unless they are carefully designed and supplemented. The magnitude of risk depends on the type and restrictiveness of the diet, the child’s age, feeding route, duration of treatment, food variety, baseline nutritional status, supplement composition and adherence, and concomitant antiseizure medication. More restrictive regimens generally allow fewer nutrient-rich foods and therefore require closer nutritional supervision [43,45,46].

Although broader safety monitoring is an essential component of ketogenic dietary therapy, the present review focuses specifically on inadequate nutrient intake, biochemical nutrient deficiencies, and impaired growth associated with dietary restriction.

#### 3.4.1. Major Nutritional Risks

The exclusion or substantial restriction of cereals, fruits, legumes, dairy products, and several vegetables may reduce the dietary supply of folate, thiamine, vitamin C, calcium, magnesium, phosphorus, and selected trace elements. The exact pattern of inadequacy varies according to the ketogenic regimen, permitted foods, age, feeding route, and composition of the prescribed supplements. Because carbohydrate-free multivitamin and mineral preparations do not always provide sufficient amounts of every nutrient, dietary intake and supplement composition should be reviewed together rather than assuming that supplementation guarantees nutritional adequacy [45,46].

Selenium is one of the most clinically important trace elements of concern. Several foods restricted during ketogenic therapy, including cereals and some protein-containing foods, ordinarily contribute to selenium intake, while many high-fat foods provide relatively little selenium. An early pediatric report identified biochemical selenium deficiency in approximately 20% of tested children and described a case of reversible cardiomyopathy associated with severe deficiency. Importantly, low concentrations developed in some patients within several months of dietary treatment rather than exclusively after prolonged exposure. Subsequent prospective evidence demonstrated a decline in plasma selenium during the first year of therapy even in children receiving supplementation, while longer-term cohorts continued to report incident deficiency [44,47,48,49].

These findings support assessment of selenium intake and periodic measurement of serum or plasma selenium, particularly in children receiving highly restrictive diets, those undergoing prolonged treatment, children with poor supplement adherence or gastrointestinal disease, and patients presenting with unexplained fatigue, muscle weakness, hair or nail changes, or cardiac symptoms. However, the available evidence is insufficient to define a single universal testing interval or supplementation dose for every child. Supplementation should therefore be individualized, and laboratory results should be interpreted using age- and laboratory-specific reference intervals.

Carnitine represents a more selective and multifactorial nutritional concern during ketogenic dietary therapy. Carnitine is required for mitochondrial transport and oxidation of long-chain fatty acids and may therefore become particularly relevant during a diet that relies heavily on fat metabolism. Low total or free carnitine concentrations have been documented during treatment, although clinically significant deficiency appears less frequent than biochemical reduction. Risk may be increased by young age, prolonged therapy, poor dietary intake, underlying metabolic disease, and concurrent treatment with valproate. In one long-term pediatric cohort, two children developed low carnitine concentrations during follow-up, although both cases were isolated and the overall prevalence remained low [44,45].

Reduced carnitine concentrations should not automatically be attributed to inadequate intake because increased reliance on fatty-acid oxidation, underlying disease, age, and medication use may also contribute. Total and free carnitine may therefore be measured at baseline and selectively during follow-up according to the indication for ketogenic therapy, medication profile, institutional protocol, previous results, and clinical presentation. Possible manifestations of clinically relevant deficiency include muscle weakness, hypotonia, lethargy, reduced appetite, and vomiting. Routine empirical supplementation of every child is not supported by the available evidence and is more appropriately considered when concentrations are low or when clinical risk is substantial [50,51].

Vitamin D and bone-related minerals require particular attention. Reduced intake of dairy products and other carbohydrate-containing foods can limit calcium, phosphorus, and magnesium intake, while vitamin D status may additionally be affected by limited dietary sources, low sunlight exposure, reduced mobility, and antiseizure medication. In a dietary study of 39 children, folate, calcium, and magnesium intake was inadequate in all participants before supplementation, and calcium, phosphorus, and magnesium frequently remained insufficient after supplementation had begun. Other pediatric cohorts have reported low vitamin D concentrations, hypocalcemia, and isolated magnesium deficiency during treatment [46,49].

The effects of ketogenic dietary therapy on skeletal health are multifactorial. In addition to inadequate intake of vitamin D and bone-related minerals, reduced mobility, underlying neurological disease, and antiseizure medication may contribute to impaired mineralization. Serum calcium alone is an insensitive indicator of nutritional adequacy because it is tightly regulated and may remain within the reference interval despite chronically inadequate calcium intake. Assessment should therefore combine dietary calcium evaluation with serum 25-hydroxyvitamin D and, when clinically indicated, phosphate, alkaline phosphatase, and parathyroid hormone [52,53].

Adverse skeletal outcomes are not inevitable. In a five-year prospective study of children with glucose transporter type 1 deficiency syndrome, growth indices and bone mineral density remained stable or increased appropriately with age. The diet was normocaloric, individually formulated, and accompanied by multivitamin and mineral supplementation, calcium, vitamin D, and an alkalinizing agent. These findings demonstrate that structured dietary treatment and close nutritional follow-up can mitigate skeletal risk, although the small and highly selected cohort does not exclude deficiencies in other pediatric populations [54].

Growth is a central longitudinal indicator of whether energy, protein, and micronutrient requirements are being met during ketogenic dietary therapy. Several pediatric cohorts suggest that linear growth may be more vulnerable than body weight. During two years of treatment, Armeno et al. [49] observed growth deceleration in a minority of children despite close follow-up, while Ruiz Herrero et al. [44] reported a significant reduction in height Z-score after two years. More recently, Bartoszewicz et al. [55] observed a decline of more than one height Z-score unit during the first year in a substantial proportion of the studied children, despite relatively stable body weight [44,49,55].

These findings illustrate why body mass index should not be used as the sole indicator of nutritional status. BMI may remain stable or temporarily increase when height velocity declines, potentially creating a misleading impression of adequate growth. Serial weight-for-age, height-for-age, and BMI-for-age Z-scores should therefore be evaluated separately and interpreted together with energy and protein intake, pubertal development, mobility, neurological severity, and adherence to the prescribed diet.

#### 3.4.2. Supplementation and Dietary Quality

A carbohydrate-free or very-low-carbohydrate multivitamin and mineral supplement is generally necessary because ketogenic diets rarely provide all micronutrients in sufficient amounts through food alone. Nevertheless, the use of a supplement does not guarantee adequacy. Commercial preparations may contain insufficient calcium, phosphorus, magnesium, selenium, or other nutrients, while formulations designed for the general pediatric population may not correspond to the specific nutritional limitations of ketogenic therapy [56,57]. Prudencio et al. [46] showed that supplementation corrected several B-vitamin inadequacies but did not consistently provide sufficient calcium, phosphorus, and magnesium, particularly in older children and adolescents.

Supplement prescriptions should therefore be reviewed according to age, sex, dietary intake, ketogenic formulation, feeding route, medication use, and laboratory findings. The nutrient composition of the complete regimen, including foods, formulas, and supplements, should be assessed rather than considering each component separately. Changes in the child’s age, body size, pubertal status, or prescribed ketogenic ratio may alter nutrient requirements and make a previously adequate supplementation regimen insufficient.

Dietary quality remains important even within the limitations imposed by ketogenic therapy. Nutrient-dense permitted foods, including nuts, seeds, avocado, oily fish, dairy products, eggs, and low-carbohydrate vegetables where compatible with the prescribed regimen, may improve the supply of essential fatty acids, high-quality protein, vitamins, minerals, and trace elements. Adequate energy and protein provision must be maintained to support growth, while unnecessary energy restriction should be avoided unless specifically indicated. Particular attention is required in infants, tube-fed children, selective eaters, and children receiving highly restrictive classic ketogenic regimens [46,58,59].

#### 3.4.3. Laboratory Assessment and Monitoring

Nutritional assessment should begin before dietary initiation so that pre-existing deficiencies, poor dietary intake, and growth abnormalities are not incorrectly attributed to ketogenic dietary therapy. The baseline evaluation should document weight, height, BMI and corresponding Z-scores, recent growth velocity, dietary intake, feeding route, food allergies or additional restrictions, supplement use, antiseizure medication, and relevant clinical symptoms.

Follow-up should be more intensive during initiation and the first year because inadequate intake, declining micronutrient concentrations, and changes in growth may become apparent during this period. Subsequent monitoring should be individualized according to age, duration and restrictiveness of the diet, feeding route, supplement composition and adherence, previous laboratory findings, growth trajectory, and clinical presentation. No single nutritional laboratory panel or testing interval is appropriate for every child receiving ketogenic dietary therapy (Table 4) [60].

Serum or plasma selenium, serum 25-hydroxyvitamin D, and selected markers of bone-mineral metabolism are particularly relevant to the nutritional focus of monitoring. Total and free carnitine should be assessed according to baseline status, medication use, underlying disease, previous results, and clinical risk rather than treated as obligatory markers in every asymptomatic child. Other vitamins and trace elements should be measured selectively when dietary assessment, supplement composition, symptoms, or previous laboratory findings indicate a plausible risk [61].

Dietary records remain essential because inadequate nutrient intake may precede a detectable biochemical abnormality, whereas normal laboratory results do not establish that the diet is nutritionally optimal. Conversely, an isolated abnormal concentration should not automatically be attributed to the ketogenic diet without considering age, inflammation, medication, underlying disease, recent supplementation, and analytical variability. Laboratory findings should therefore always be interpreted together with dietary intake, supplement adherence, growth, and clinical examination.

Ketogenic dietary therapy carries a genuine risk of nutritional inadequacy because of the marked restriction of several nutrient-rich food groups, but deficiencies and impaired growth are not inevitable. Individualized diet formulation, adequate energy and protein provision, appropriate supplementation, assessment of selenium, vitamin D, and bone-mineral status, selective evaluation of carnitine, and longitudinal monitoring of growth can substantially reduce this risk. The objective of nutritional monitoring is to identify inadequate intake or declining nutrient status before clinically significant deficiency or growth impairment develops. Ketogenic dietary therapy can be implemented safely in many children when it is individually formulated, accompanied by appropriate supplementation, and monitored by an experienced multidisciplinary team [62,63].

### 3.5. Therapeutic Diets for Inherited Metabolic Disorders: Phenylketonuria as the Principal Model and Selected Examples

Dietary therapy is a central component of treatment for many inherited metabolic disorders (IEMs), particularly those affecting amino acid and organic acid metabolism. Unlike elective restrictive diets, these regimens are medically prescribed to limit the intake of substrates that cannot be adequately metabolized, reduce the accumulation of toxic metabolites, and prevent neurological or systemic complications. Treatment may involve restriction of natural protein or specific amino acids, use of specialized medical formulas, and targeted supplementation. Because dietary therapy often begins in infancy and continues throughout life, it must achieve metabolic control while simultaneously providing sufficient energy, protein, essential amino acids, vitamins, minerals, and fatty acids to support normal growth and development [64,65].

Satisfactory biochemical control of the underlying disorder does not necessarily confirm nutritional adequacy. A child may maintain the target concentration of a disease-specific metabolite while consuming insufficient natural protein, protein substitute, energy, micronutrients, or essential fatty acids. Conversely, fortified medical formulas and additional supplements may result in high intakes of selected nutrients [2,66,67]. Nutritional assessment should therefore be conducted alongside, but separately from, disease-specific biochemical monitoring.

The nutritional consequences of therapeutic diets vary according to the metabolic defect, the nutrient being restricted, the permitted intake of natural protein, the composition of the medical formula, age, growth phase, treatment adherence, and the use of pharmacological therapies [2,66,68,69]. Phenylketonuria is used as the principal model because its lifelong dietary management and nutritional outcomes have been extensively studied. Hereditary tyrosinemia type I, organic acidurias, and urea-cycle disorders are included as selected examples of related nutritional challenges rather than as a comprehensive review of dietary treatment for all IEMs [70,71,72,73].

Disease-specific metabolites remain essential for treatment adjustment and the prevention of metabolic decompensation. However, target concentrations of these metabolites do not by themselves establish nutritional adequacy and should be interpreted together with dietary intake, growth, clinical findings, and relevant nutritional biomarkers [1,2,6,7,9,10].

#### 3.5.1. General Nutritional Considerations in Protein-Restricted IEM Diets

Many therapeutic diets for amino acid and organic acid disorders restrict natural protein or selected amino acids and therefore rely on specialized amino acid mixtures or protein substitutes to meet protein and micronutrient requirements [2,66,68]. Because these products are commonly fortified, inadequate intake may reduce the supply of essential amino acids as well as iron, vitamin B12, folate, zinc, selenium, calcium, vitamin D, and other micronutrients [2,67,68]. Adherence is particularly challenging during adolescence because of the taste and volume of protein substitutes, treatment burden, social influences, and increasing independence in food selection [66,69].

Protein restriction should be individualized and should not exceed the level required for metabolic control. Excessive restriction or inadequate energy intake may contribute to essential amino acid deficiency, catabolism, loss of lean tissue, and impaired growth. Dietary prescriptions should therefore be regularly adjusted according to age, body weight, growth, metabolic tolerance, clinical status, and biochemical response [66,70,71].

Serum albumin and prealbumin should not be used alone to assess protein adequacy because they are strongly influenced by inflammation, hepatic function, renal losses, hydration, and disease severity [74]. Dietary intake, consumption and distribution of the prescribed protein substitute, serial growth assessment, and plasma amino acid profiles, when clinically indicated, provide a more informative evaluation of long-term nutritional adequacy [66,70,71,74].

#### 3.5.2. Phenylketonuria

Phenylketonuria (PKU) is the best-studied example of a lifelong therapeutic restrictive diet. Conventional treatment limits natural protein to reduce phenylalanine intake while providing protein requirements through phenylalanine-free amino acid mixtures or other specialized protein substitutes. Measured amounts of phenylalanine-containing foods, fruits, vegetables, and low-protein products complete the diet. The degree of dietary restriction depends on residual phenylalanine hydroxylase activity, metabolic tolerance, age, growth, treatment response, and the use of pharmacological therapies such as sapropterin [64,75].

Although the traditional PKU diet is predominantly plant-based, it is more restrictive than conventional vegetarian or vegan diets because, in addition to most animal-protein sources, many protein-rich plant foods, including legumes, nuts, and soy products, are also restricted [75,76]. Consequently, phenylalanine-free or low-phenylalanine protein substitutes usually provide most of the protein requirement and are commonly fortified with vitamins and minerals; some formulations also contain essential fatty acids or long-chain polyunsaturated fatty acids. Nutritional adequacy therefore depends on the prescribed amount and composition of the protein substitute, adherence to its regular consumption, natural-protein tolerance, the quality of the permitted diet, and any additional supplementation [76,77].

Maintaining adequate protein and essential amino acid intake remains a major nutritional priority throughout childhood and adolescence. Although protein intake may appear sufficient when calculated as protein equivalents, free amino acid mixtures are absorbed more rapidly and may be utilized less efficiently than intact dietary protein. Protein requirements in PKU are therefore generally prescribed above standard population requirements, particularly when amino acid-based substitutes provide most of the protein intake. The prescribed protein substitute should also be divided into three or four doses distributed throughout the day, preferably consumed with natural protein and an energy source, to support protein synthesis and metabolic control [76,78].

Plasma phenylalanine primarily reflects metabolic control rather than nutritional adequacy. When poor adherence, excessive natural-protein restriction, or inadequate protein-substitute intake is suspected, measurement of tyrosine and the broader plasma amino acid profile may provide additional information. Laboratory findings should always be interpreted in the context of recent dietary intake, formula consumption, illness, and overall metabolic stability. Growth monitoring and dietary assessment remain indispensable because isolated biochemical measurements cannot reliably confirm long-term protein adequacy. Earlier studies reported impaired growth in some children with PKU, whereas more recent cohorts generally demonstrate normal growth when energy, protein, and micronutrient requirements are consistently achieved [79].

Iron, vitamin B12, folate, zinc, and selenium are the micronutrients most frequently considered in children with PKU because their principal natural dietary sources are substantially restricted. In well-treated patients, fortified protein substitutes usually provide sufficient amounts of these nutrients; however, deficiencies may occur when formula adherence is poor or intake is inadequate.

Iron status should be evaluated using a complete blood count together with ferritin, while inflammatory markers such as C-reactive protein may assist in interpreting ferritin concentrations when inflammation is suspected. Serum iron alone has limited diagnostic value because of its considerable biological variability. Vitamin B12 and folate assessment is particularly relevant in patients with poor adherence to medical formula, prolonged restriction of natural protein, macrocytosis, or previous evidence of deficiency. Conversely, elevated circulating vitamin B12 concentrations may reflect the use of highly fortified protein substitutes or additional supplementation rather than pathological conditions.

Published studies demonstrate variable findings regarding zinc and selenium status. While deficiencies have been reported in subsets of children, many patients who consistently consume prescribed protein substitutes maintain concentrations within reference ranges. Similarly, studies have described occasional deficiencies of folate, vitamin D, zinc, or selenium alongside elevated vitamin B12 concentrations, emphasizing that laboratory abnormalities depend largely on dietary adherence, formula composition, supplementation practices, and local dietary patterns rather than representing universal features of PKU [80,81]. These findings support individualized laboratory assessment according to dietary intake, symptoms, previous laboratory abnormalities, and clinical risk factors instead of routine measurement of all micronutrients in every patient.

Bone health requires ongoing attention because the traditional PKU diet restricts many dairy and other natural protein sources. Reduced formula intake, inadequate calcium consumption, insufficient vitamin D supplementation, and low physical activity may contribute to impaired bone mineralization. Serum calcium is a poor indicator of calcium adequacy because circulating concentrations are tightly regulated. Assessment should therefore emphasize dietary calcium intake, adherence to protein substitutes and supplements, serum 25-hydroxyvitamin D, growth, physical activity, and fracture history. Measurement of phosphate, alkaline phosphatase, and parathyroid hormone may be appropriate when deficiency or impaired mineralization is suspected, while bone densitometry should be reserved for selected patients with persistent clinical risk factors rather than performed routinely.

Long-chain polyunsaturated fatty acids, particularly docosahexaenoic acid (DHA), represent another potential nutritional concern because fish and other animal-derived foods are substantially restricted. Some protein substitutes are supplemented with DHA, whereas others are not. Routine measurement of plasma or erythrocyte fatty acid composition is not required for all patients, but dietary intake and formula composition should be reviewed regularly to ensure an adequate source of DHA. Laboratory assessment may be considered in children with poor formula adherence, highly restrictive diets, formulas lacking long-chain polyunsaturated fatty acids, or specific neurological or nutritional concerns [75,82].

#### 3.5.3. Selected Other Protein-Restricted Disorders

Although PKU provides the most extensively studied model, related nutritional challenges occur in other disorders treated by restriction of natural protein or selected amino acids. However, dietary prescriptions, protein substitutes, supplementation, and biochemical monitoring must remain disorder-specific.

##### Hereditary Tyrosinemia Type I

In hereditary tyrosinemia type I, nitisinone inhibits tyrosine degradation upstream of the primary enzymatic defect, thereby preventing the formation of toxic metabolites but increasing circulating tyrosine. Treatment therefore combines nitisinone with restriction of tyrosine and its precursor phenylalanine, usually supported by a phenylalanine- and tyrosine-free amino acid mixture.

The nutritional objective is to control tyrosine concentrations while maintaining adequate phenylalanine availability and normal growth. Excessive restriction of natural protein may lead to persistently low plasma phenylalanine. This should prompt reassessment of natural-protein intake and the dietary prescription; phenylalanine supplementation is used in some centres, although its timing and dosage are not fully standardized and it may increase plasma tyrosine. Monitoring should include natural-protein intake, adherence to the amino acid mixture, growth, and plasma tyrosine and phenylalanine concentrations [72,83].

##### Methylmalonic and Propionic Acidemias

Long-term dietary management of methylmalonic and propionic acidemias is based on individualized restriction of precursor amino acids through controlled natural-protein intake, together with sufficient energy to prevent fasting and catabolism. Precursor-free amino acid mixtures may be used when tolerated natural protein is insufficient to meet protein requirements, but excessive reliance on these products may contribute to an imbalanced amino acid supply.

Nutritional assessment should include natural- and total-protein intake, use of medical formula, energy intake, feeding tolerance, growth, and plasma amino acid concentrations. Carnitine supplementation and monitoring are particularly relevant in these disorders because secondary depletion and increased acylcarnitine formation may occur. Plasma free, total, and acylcarnitine concentrations may therefore help guide treatment, but carnitine should not be regarded as a universal nutritional marker for all inherited metabolic disorders [70,84].

##### Urea-Cycle Disorders

Chronic management of urea-cycle disorders generally combines individualized protein restriction with sufficient energy intake to prevent endogenous protein catabolism. Essential amino acid supplements may be required when natural-protein tolerance is too low to support growth and metabolic stability. Nutritional monitoring should include total and natural-protein intake, energy intake, supplement use, growth, clinical status, and plasma amino acid profiles, with interpretation adapted to the specific urea-cycle defect and pharmacological treatment [73].

Disease-specific biochemical parameters remain indispensable for preventing metabolic decompensation, but they primarily evaluate metabolic control rather than nutritional deficiency and are therefore outside the main scope of this review.

#### 3.5.4. Laboratory Assessment and Monitoring

Nutritional monitoring should be individualized according to the specific disorder, degree of natural-protein restriction, composition and intake of the prescribed medical formula, age, growth, treatment adherence, pharmacological therapy, clinical findings, and previous laboratory results [2,66,68]. No single nutritional laboratory panel is appropriate for all inherited metabolic disorders because dietary prescriptions and associated nutritional risks differ substantially between conditions (Table 5) [2,68,70,73,84].

In children receiving protein-restricted diets, follow-up should combine serial assessment of growth, detailed dietary review, evaluation of medical-formula adherence, and targeted laboratory testing for nutrients plausibly affected by dietary restriction or inadequate formula intake. Disease-specific biochemical markers remain essential for evaluating metabolic control, but they do not replace assessment of overall nutritional adequacy. The approach proposed below is therefore intended primarily for PKU and selected protein-restricted amino acid and organic acid disorders and should not be interpreted as a universal protocol for all IEMs. Therapeutic diets for inherited metabolic disorders are medically essential and can support normal growth when natural-protein restriction, medical-formula intake, energy provision, and supplementation are adjusted regularly according to metabolic control and nutritional status. [2,66,68,70,73,84].

### 3.6. Shared and Condition-Specific Principles Across Restrictive Diets

Although the dietary patterns included in this review differ substantially in indication, composition, and clinical context, several assessment principles are common to all. Nutritional risk is determined more reliably by the foods or nutrients actually excluded, the adequacy of replacement foods, use of fortified products, supplements or medical formulas, adherence, duration of restriction, and the child’s growth phase than by the dietary label alone. Across all dietary patterns, dietary history, longitudinal anthropometry, clinical examination, and review of supplementation should precede laboratory testing, while biomarkers should be selected only when a plausible nutrient gap or clinical concern is present.

At the same time, the relevant nutritional risks remain condition specific. Vitamin B12 is a central concern in vegan diets; calcium, vitamin D, iodine, protein, and energy require particular attention after cow’s-milk or multiple-food elimination; iron, folate, B vitamins, and fiber depend strongly on the quality of gluten-free replacement foods; selenium, vitamin D, bone-related minerals, selected carnitine assessment, and linear growth are especially relevant during ketogenic dietary therapy; and protein-substitute adherence, essential amino acids, and selected micronutrients are central in phenylketonuria and related protein-restricted disorders. The shared framework therefore guides the structure of assessment, whereas the selection of individual biomarkers remains diet- and condition-specific.

## 4. Interpretation of Nutritional Biomarkers and Individualized Follow-Up

**Figure 1:**
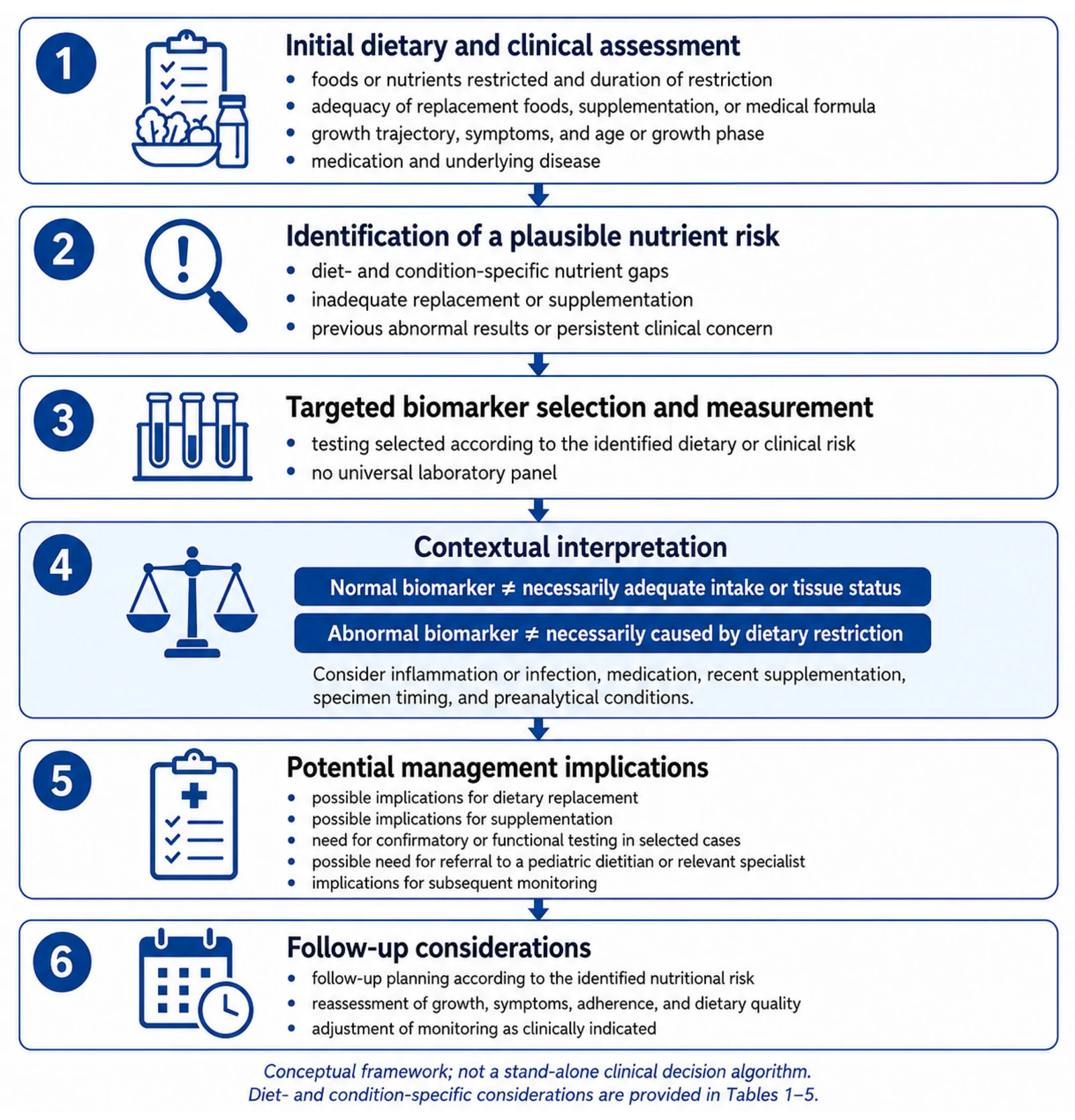
Conceptual risk-based framework for the interpretation of nutritional biomarkers and individualized follow-up in children following restrictive diets. The framework summarizes the common sequence of identifying a plausible nutrient gap, evaluating dietary replacement and supplementation, assessing growth and clinical risk, selecting targeted biomarkers, interpreting results in their clinical and analytical context, and determining individualized management and follow-up. It is not intended as a universal or stand-alone clinical decision algorithm. Diet-specific assessments, testing considerations, and interpretative limitations are provided in Table 1, Table 2, Table 3, Table 4 and Table 5.

Laboratory biomarkers should be interpreted within a structured clinical sequence rather than as isolated indicators of nutritional adequacy. The first step is to identify the exact foods or nutrients restricted and determine whether appropriate replacement foods, fortified products, supplements, or medical formulas are consistently used. These findings should then be considered together with longitudinal growth, dietary intake, symptoms, developmental stage, and the underlying condition to decide whether laboratory testing is justified. When testing is performed, results must be interpreted in relation to inflammation, infection, medication, organ function, recent supplementation, and preanalytical variation. A result within the reference interval does not always confirm adequate intake or tissue status, whereas an isolated abnormal result may reflect factors other than dietary deficiency. History-taking and physical examination should be systematic and adapted to the child’s age and developmental stage. In infants and younger children, relevant information may depend largely on caregiver observation and should include feeding behavior, food selectivity, swallowing difficulties, pica, changes in activity, and observed changes in the hair or skin. Older children and adolescents may additionally report hair loss or changes in hair pigmentation, night blindness, anosmia or dysgeusia, dysphagia, pica-particularly pagophagia-rashes, or pruritus. Physical examination should include assessment of growth and general appearance; hair density, growth, and pigmentation; the lips, oral mucosa, and tongue for cheilitis, angular cheilosis, or glossitis; the skin for rashes or periorificial dermatitis; cardiac rate and rhythm; and neurological findings such as ataxia, hyporeflexia, or areflexia. These findings are not specific to a single nutrient deficiency and may reflect combined deficiencies or other medical conditions. They should therefore be interpreted together with dietary history, growth, supplementation, and the underlying condition and used to guide targeted rather than indiscriminate laboratory testing. Laboratory findings should be interpreted using age-, sex-, pubertal-stage-, assay-, and laboratory-specific reference intervals where available. C-reactive protein may assist in interpreting ferritin when inflammation is clinically plausible or when ferritin appears normal or elevated despite suspected iron deficiency. Transferrin saturation or soluble transferrin receptor may be useful when ferritin and hematological findings are discordant. Methylmalonic acid and/or homocysteine may be considered when vitamin B12 is low, borderline, or inconsistent with hematological, developmental, or neurological findings. Borderline or unexpected results should generally be reassessed after reviewing recent supplementation, fasting status, specimen timing, acute illness, and relevant preanalytical conditions. Repeat testing should be individualized according to the severity of the abnormality, persistence of nutritional risk, treatment introduced, symptoms, and growth trajectory rather than assigned a universal interval [2,66]. Figure 1 presents a conceptual framework for risk-based interpretation of nutritional biomarkers and individualized follow-up. It is intended to support the general sequence of clinical reasoning and should not be interpreted as a stand-alone or validated clinical decision algorithm. Diet- and condition-specific nutritional risks and assessment considerations are presented in Section 3.1, Section 3.2, Section 3.3, Section 3.4 and Section 3.5 and Table 1, Table 2, Table 3, Table 4 and Table 5.

In practical terms, the conceptual framework comprises six linked considerations: (1) initial dietary and clinical assessment, including the foods or nutrients restricted, adequacy of replacement foods, supplementation or medical-formula use, growth trajectory, symptoms, medication, and underlying disease; (2) identification of a plausible diet- or condition-specific nutrient risk; (3) selection and measurement of biomarkers addressing the identified dietary or clinical concern; (4) interpretation of laboratory findings in relation to inflammation, infection, medication, recent supplementation, specimen timing, and preanalytical conditions; (5) consideration of potential implications for dietary replacement, supplementation, confirmatory testing, specialist referral, and subsequent monitoring; and (6) follow-up planning according to the persistence and clinical significance of the identified nutritional risk. These considerations are intended to support individualized clinical reasoning rather than establish universal laboratory panels, fixed monitoring intervals, or prescriptive management pathways. Several common examples illustrate these limitations. Ferritin may increase during inflammation, and iron depletion may precede the development of anemia or microcytosis [39,85]. Similarly, vitamin B12 deficiency should not be excluded solely because anemia or macrocytosis is absent [86]. Serum calcium is tightly regulated and does not reliably reflect dietary calcium intake, while albumin and prealbumin primarily reflect inflammation and disease severity rather than body protein stores [74,87]. Plasma zinc is also influenced by inflammation, fasting status, time of sampling, and preanalytical conditions [88].

Follow-up should therefore be individualized according to age, growth, severity and duration of dietary restriction, adequacy of dietary replacement, adherence, previous deficiencies, clinical findings, and persistence of nutritional risk. Repeat testing is particularly appropriate after a deficiency has been identified, supplementation has been introduced or modified, symptoms or growth abnormalities persist, or the restrictive diet continues to create a plausible nutrient gap [2,66].

## 5. Future Perspectives and Conclusions

Restrictive dietary patterns may support normal growth and development when they are appropriately indicated or selected, age-appropriate, nutritionally balanced, and monitored according to the type and severity of restriction, the child’s age and growth phase, the underlying disease, and the need for specialist supervision. However, children have specific nutritional requirements related to growth and development, making them particularly vulnerable to deficiencies when key foods or nutrients are excluded without adequate replacement. Risk-based laboratory assessment, interpreted together with dietary intake, supplementation, growth, and clinical findings, can support the early identification of nutritional inadequacies before clinically significant deficiency develops. The purpose of nutritional monitoring is therefore not to characterize restrictive diets as inherently harmful, but to identify modifiable gaps in dietary replacement, supplementation, or treatment adherence before clinically significant deficiency develops.

Future nutritional monitoring may increasingly incorporate metabolomic profiling, digital assessment of growth and dietary intake, and AI-assisted interpretation of complex clinical and laboratory data. These approaches are presented as forward-looking possibilities rather than as conclusions of a systematic evaluation within the present review. Their clinical value will require validation in pediatric populations, standardized data collection, transparent decision-support methods, and continued interpretation by multidisciplinary healthcare teams. This review has limitations inherent to narrative synthesis. No formal risk-of-bias assessment or evidence grading was performed, and heterogeneity in dietary definitions, age groups, supplementation practices, underlying conditions, laboratory methods, and reported outcomes limits the strength of universal monitoring conclusions.

## Figures and Tables

**Table 1:** Proposed risk-based nutritional assessment in children following plant-based diets.

Nutritional Concern	Potential Assessment According to Identified Risk	Main Interpretative Considerations
Vitamin B12	Initial: serum or plasma vitamin B12 or holotranscobalamin; CBC and MCV. When indicated: methylmalonic acid and/or homocysteine for low, borderline, or discordant results	Normal CBC or MCV does not exclude functional deficiency. Recent supplementation may produce high circulating vitamin B12 concentrations without necessarily reflecting long-term adequacy.
Iron status	Initial: CBC, hemoglobin, MCV, RDW, and ferritin. When indicated: CRP when inflammation is suspected; transferrin saturation or soluble transferrin receptor in uncertain or discordant cases	Ferritin is an acute-phase reactant and may increase during inflammation. Depleted iron stores may precede anemia. Serum iron alone has limited diagnostic value.
Vitamin D and bone-related minerals	Initial: serum 25-hydroxyvitamin D when dietary intake, supplementation, sunlight exposure, growth, or clinical history indicates increased risk. When indicated: calcium, phosphate, alkaline phosphatase, and PTH in children with deficiency, poor calcium intake, impaired growth, bone pain, fractures, or other skeletal concerns	Normal serum calcium does not demonstrate adequate calcium intake or normal bone mineralization. Interpretation of 25-hydroxyvitamin D should consider season, sunlight exposure, skin pigmentation, supplementation, and assay-related variation.
Iodine and thyroid function	Initial: dietary assessment of iodized salt, fortified foods, seaweed products, and iodine-containing supplements. When indicated: TSH and free T4 in children with clinical or dietary risk; urinary iodine in selected specialist assessments	Spot urinary iodine reflects recent intake and is more informative at the population level than for diagnosing deficiency in an individual child. Thyroid-function tests are not direct measures of dietary iodine intake.
Zinc	Initial: dietary assessment. When indicated: serum or plasma zinc when deficiency is clinically suspected or dietary intake is markedly limited	Circulating zinc is influenced by inflammation, fasting status, sampling time, recent meals, and preanalytical conditions. A normal concentration does not necessarily exclude marginal intake.
Selenium	Initial: dietary assessment, including food origin and supplement use. When indicated: serum or plasma selenium in selected high-risk children	Selenium intake varies geographically according to soil content and food origin. Available evidence does not support routine biochemical screening in all children following plant-based diets.
Protein and energy adequacy	Initial: detailed dietary assessment and longitudinal evaluation of weight, height, and BMI-for-age Z-scores. When indicated: albumin, prealbumin, or total protein only when substantial nutritional compromise or another clinical indication is present	Circulating proteins are late and nonspecific markers of inadequate protein intake and are influenced by inflammation, liver function, renal losses, and hydration. Adequate calculated protein intake does not necessarily confirm adequate amino acid quality or total energy intake.
Long-chain omega-3 fatty acids	Initial: dietary assessment of alpha-linolenic acid and direct DHA sources, including fortified foods or algal supplements. When indicated: plasma or erythrocyte fatty-acid profile in specialist or research settings	Alpha-linolenic acid intake does not necessarily ensure adequate EPA or DHA status because endogenous conversion is limited. Routine biochemical testing is not established for otherwise healthy children.
Other potential micronutrient inadequacies	Initial: review of dietary variety, fortified foods, and supplements. When indicated: targeted testing for nutrients such as riboflavin, vitamin A, or choline when a plausible dietary or clinical risk is identified	Evidence is insufficient to support universal biochemical screening. Testing should correspond to the specific foods excluded, symptoms, and documented dietary inadequacy.

Note: The assessments presented in this table reflect condition-specific professional guidance where available. Where formal recommendations are limited, they represent the authors’ qualitative synthesis of pediatric evidence and established principles of nutritional biomarker interpretation. They are not intended as mandatory screening panels or fixed monitoring protocols.

**Table 2:** Proposed risk-based nutritional assessment in children following food-allergy elimination diets.

Nutritional Concern	Potential Assessment According to Identified Risk	Main Interpretative Considerations
Iron status	Initial: CBC, hemoglobin, MCV, RDW, and ferritin when iron-rich foods or fortified products are substantially restricted. When indicated: CRP when inflammation is suspected; transferrin saturation or soluble transferrin receptor in uncertain or discordant cases	Ferritin is an acute-phase reactant and may increase during inflammation. Iron depletion may precede anemia. Serum iron alone is not sufficient for assessment of iron status.
Vitamin D status	Initial: serum 25-hydroxyvitamin D in children with prolonged milk elimination, low dietary intake, inconsistent supplementation, limited sunlight exposure, impaired growth, or skeletal symptoms	Interpretation should consider season, sunlight exposure, skin pigmentation, obesity, supplementation, national fortification practices, and assay-related variation.
Calcium and bone-related minerals	Initial: dietary assessment of calcium intake, including fortified substitutes and supplements. When indicated: calcium, phosphate, alkaline phosphatase, and PTH in children with poor intake, low 25-hydroxyvitamin D, impaired growth, bone pain, fractures, or prolonged unsupervised milk elimination	Normal serum calcium does not demonstrate adequate calcium intake or normal bone mineralization because circulating calcium is tightly regulated.
Protein and energy adequacy	Initial: detailed dietary assessment and longitudinal evaluation of weight, height, and BMI-for-age Z-scores. When indicated: albumin, total protein, or prealbumin only when substantial nutritional compromise or another clinical indication is present	Circulating proteins are nonspecific and are influenced by inflammation, hepatic synthesis, renal losses, and hydration. Growth and dietary intake are more informative for detecting chronic inadequacy.
Vitamin B12 and folate	Initial: dietary assessment based on the foods excluded and the composition of substitutes. When indicated: serum vitamin B12 and folate in children avoiding several animal-source foods, those with macrocytosis, anemia, neurological symptoms, poor dietary variety, or additional restrictions	These nutrients are not universally affected by food-allergy diets. Testing should reflect the actual foods excluded and the nutritional adequacy of replacements.
Zinc and selenium	Initial: dietary assessment. When indicated: serum or plasma zinc or selenium in children with multiple-food elimination, poor growth, highly limited dietary variety, compatible symptoms, or previous abnormal results	Circulating concentrations are influenced by inflammation and preanalytical factors. Selenium intake also varies according to geographical food composition. Routine universal testing is not supported.
Iodine and thyroid function	Initial: dietary assessment in children avoiding cow’s milk, fish, eggs, iodized salt, or fortified products. When indicated: TSH and free T4 when clinical concern is present; urinary iodine in selected specialist assessments	Iodine risk depends strongly on regional food composition and fortification practices. Spot urinary iodine is more useful for population assessment than for diagnosing deficiency in an individual child.
Other potential micronutrient inadequacies	Initial: assessment based on the number and nutritional importance of foods excluded, substitute products, supplementation, and dietary variety. When indicated: targeted testing for specific nutrients according to a plausible dietary or clinical risk	No universal laboratory panel is appropriate for all children with food allergy. Testing should correspond to the nutrients normally supplied by the excluded foods.

Note: The assessments presented in this table reflect condition-specific professional guidance where available. Where formal recommendations are limited, they represent the authors’ qualitative synthesis of pediatric evidence and established principles of nutritional biomarker interpretation. They are not intended as mandatory screening panels or fixed monitoring protocols.

**Table 3:** Proposed risk-based nutritional assessment in children following a gluten-free diet.

Nutritional Concern	Potential Assessment According to Identified Risk	Main Interpretative Considerations
Iron status	CBC, hemoglobin, MCV, RDW, ferritin	Ferritin should be interpreted with CRP or clinical inflammatory status; iron depletion may occur before anemia
Folate status	Serum folate	Low dietary intake may precede biochemical deficiency; interpret with dietary history and supplementation
Vitamin B12 status	Serum vitamin B12	MMA or homocysteine may be considered when results are borderline or symptoms are suggestive
Vitamin D status	Serum 25(OH)D	Consider season, sunlight exposure, skin pigmentation, obesity, supplementation, and local fortification
Bone/mineral metabolism	Calcium, phosphate, ALP and PTH when clinically indicated	Normal serum calcium does not confirm adequate calcium intake or normal bone health
Dietary fiber	No routine biochemical marker	Assess whole-grain and plant-food intake, stool pattern, and dependence on refined gluten-free products

Note: The assessments presented in this table reflect condition-specific professional guidance where available. Where formal recommendations are limited, they represent the authors’ qualitative synthesis of pediatric evidence and established principles of nutritional biomarker interpretation. They are not intended as mandatory screening panels or fixed monitoring protocols.

**Table 4:** Proposed risk-based nutritional assessment in children following a ketogenic dietary therapy.

Nutritional Concern	Potential Assessment According to Identified Risk	Main Interpretative Considerations
Selenium status	Serum or plasma selenium	Interpret together with dietary intake and supplement composition; severe deficiency may have cardiac consequences
Carnitine status	Total and free carnitine, with acyl/free carnitine ratio when indicated	Particularly relevant with valproate use, prolonged therapy, young age, compatible symptoms, underlying metabolic disease, or low baseline values
Vitamin D status	Serum 25-hydroxyvitamin D	Consider season, sunlight exposure, mobility, supplementation, obesity, and antiseizure medication
Bone and mineral nutrition	Dietary calcium, phosphorus, and magnesium assessment; serum calcium, phosphate, alkaline phosphatase, and PTH when indicated	Normal serum calcium does not exclude inadequate calcium intake or impaired mineralization
General micronutrient adequacy	Dietary assessment and review of multivitamin and mineral supplement composition; targeted biochemical testing according to identified risk	Supplement use does not guarantee adequate intake; requirements and supplement composition vary according to age and ketogenic regimen
Protein and energy adequacy	Detailed dietary assessment; serial weight, height, and BMI Z-scores	Growth parameters should be evaluated separately because stable BMI may mask reduced linear growth
Other suspected deficiencies	Targeted testing based on dietary intake, clinical symptoms, and supplement composition	Routine indiscriminate testing is not supported; testing should correspond to a plausible dietary or clinical risk

Note: The assessments presented in this table reflect condition-specific professional guidance where available. Where formal recommendations are limited, they represent the authors’ qualitative synthesis of pediatric evidence and established principles of nutritional biomarker interpretation. They are not intended as mandatory screening panels or fixed monitoring protocols.

**Table 5:** Proposed risk-based nutritional assessment in children receiving therapeutic diets for phenylketonuria and selected protein-restricted inherited metabolic disorders.

Nutritional Concern	Potential Assessment According to Identified Risk	Main Interpretative Considerations
Protein and essential amino acid adequacy	Dietary natural-protein and protein-substitute intake; growth; plasma amino acid profile when indicated	Interpret in relation to the specific disorder, timing of formula intake, recent illness, metabolic stability, and prescribed treatment
Iron status	CBC, hemoglobin, MCV, RDW, ferritin; CRP or transferrin saturation when indicated	Normal hemoglobin does not exclude depleted iron stores; ferritin may increase during inflammation
Vitamin B12 and folate	Serum vitamin B12 and folate; methylmalonic acid or homocysteine for equivocal B12 results	Particularly relevant with poor formula adherence, low animal-food intake, macrocytosis, neurological symptoms, or previous abnormal results
Vitamin D and bone-related minerals	Serum 25-hydroxyvitamin D; dietary calcium assessment; calcium, phosphate, ALP, and PTH when clinically indicated	Normal serum calcium does not confirm adequate intake or normal mineralization
Zinc and selenium	Serum or plasma zinc and selenium in selected high-risk children	Interpret according to dietary restriction, formula composition, inflammation, supplement use, regional food composition, and previous findings
Long-chain polyunsaturated fatty acids	Dietary and formula assessment; plasma or erythrocyte fatty-acid profile in selected cases	Consider when DHA intake is inadequate, formula adherence is poor, or the medical formula does not contain long-chain polyunsaturated fatty acids
Carnitine in selected disorders	Total and free carnitine, with additional indices when clinically appropriate	Relevant mainly in disorders or treatments associated with secondary depletion; not a universal marker for all IEMs
Growth and overall dietary adequacy	Weight, height, BMI and corresponding Z-scores; head circumference in young children; detailed dietary assessment	Height, weight, and BMI should be interpreted separately and longitudinally; normal BMI may coexist with reduced linear growth
Other suspected deficiencies	Targeted testing according to the specific restriction, formula composition, clinical symptoms, and previous results	Routine indiscriminate micronutrient testing is not supported

Note: The assessments presented in this table reflect condition-specific professional guidance where available. Where formal recommendations are limited, they represent the authors’ qualitative synthesis of pediatric evidence and established principles of nutritional biomarker interpretation. They are not intended as mandatory screening panels or fixed monitoring protocols.

## Data Availability

No new data were created in this study. Data sharing is not applicable to this article as no datasets were generated or analyzed during the current study.

## Abbreviations

The following abbreviations are used in this manuscript:

25(OH)D	25-hydroxyvitamin D
AI	artificial intelligence
ALP	alkaline phosphatase
BMI	body mass index
CBC	complete blood count
CRP	C-reactive protein
DHA	docosahexaenoic acid
EPA	eicosapentaenoic acid
GFD	gluten-free diet
GLUT1	glucose transporter type 1
IEM	inherited metabolic disorder
MCV	mean corpuscular volume
MMA	methylmalonic acid
PKU	phenylketonuria
PTH	parathyroid hormone
RBP	retinol-binding protein
RDW	red cell distribution width
T4	thyroxine
TSH	thyroid-stimulating hormone
